# Induction of intestinal pro-inflammatory immune responses by lipoteichoic acid

**DOI:** 10.1186/1476-9255-9-7

**Published:** 2012-03-16

**Authors:** Mojgan Zadeh, Mohammad W Khan, Yong Jun Goh, Kurt Selle, Jennifer L Owen, Todd Klaenhammer, Mansour Mohamadzadeh

**Affiliations:** 1 Department of Infectious Diseases and Pathology, Emerging Pathogens Institute, and Cancer Genetic Institute, University of Florida, Gainesville, FL, USA; 2 Northwestern University, Feinberg School of Medicine, Chicago, IL, USA; 3 Department of Food, Bioprocessing & Nutrition Sciences, N.C. State University, Raleigh, NC, USA

**Keywords:** Dendritic cells, Dextran sulfate sodium, Inflammatory bowel disease, Lipoteichoic acid, Toll-like receptor 2

## Abstract

**Background:**

The cellular and molecular mechanisms of inflammatory bowel disease are not fully understood; however, data indicate that uncontrolled chronic inflammation induced by bacterial gene products, including lipoteichoic acid (LTA), may trigger colonic inflammation resulting in disease pathogenesis. LTA is a constituent glycolipid of Gram-positive bacteria that shares many inflammatory properties with lipopolysaccharide and plays a critical role in the pathogenesis of severe inflammatory responses via Toll-like receptor 2. Accordingly, we elucidate the role of LTA in immune stimulation and induced colitis in vivo.

**Methods:**

To better understand the molecular mechanisms utilized by the intestinal microbiota and their gene products to induce or subvert inflammation, specifically the effect(s) of altered surface layer protein expression on the LTA-mediated pro-inflammatory response, the *Lactobacillus acidophilus s*urface *l*ayer *p*rotein (*Slp*) genes encoding SlpB and SlpX were deleted resulting in a SlpB^- ^and SlpX^- ^mutant that continued to express SlpA (assigned as NCK2031).

**Results:**

Our data show profound activation of dendritic cells by NCK2031, wild-type *L. acidophilus *(NCK56), and purified *Staphylococcus aureus*-LTA. In contrary to the LTA-deficient strain NCK2025, the LTA-expressing strains NCK2031 and NCK56, as well as *S. aureus*-LTA, induce pro-inflammatory innate and T cell immune responses in vivo. Additionally, neither NCK2031 nor *S. aureus*-LTA supplemented in drinking water protected mice from DSS-colitis, but instead, induced significant intestinal inflammation resulting in severe colitis and tissue destruction.

**Conclusions:**

These findings suggest that directed alteration of two of the *L. acidophilus *NCFM-Slps did not ameliorate LTA-induced pro-inflammatory signals and subsequent colitis.

## Background

The intestinal immune system must co-exist with resident commensal microorganisms while maintaining the ability to defend against potential microbial challenge. This immune tolerance is a highly regulated process comprised of a myriad of biological checkpoints necessary to maintain homeostasis between the host and the gut microbiota [1]. In instances of inflammatory bowel disease (IBD), this tolerance between immune cells and intestinal bacteria is disrupted; however, causes of this tolerance breakdown have not yet been determined [2,3]. Although the etiology of IBD is still unknown, exaggerated inflammation induced by activated innate immune cells via their interaction with the microbiota and their gene products, as well as infiltrating CD4^+ ^IFNγ^+ ^T cells, likely play key roles in uncontrolled inflammation and tissue destruction [4-6]. Foxp3^+ ^regulatory T cells (Tregs) also critically control intestinal inflammation [7] and significantly prevent colitis [8], suggesting a pivotal role for Tregs in intestinal immune homeostasis [9].

A fundamental challenge in preventing an imbalanced immune response is the understanding of how the host immune system distinguishes a pathogen from normal intestinal flora. One of the commensal microorganisms of the gut is *L. acidophilus*, which expresses unique *s*urface *l*ayer *p*roteins (Slps), including A, B, X, and abundant lipoteichoic acid (LTA). LTA is a zwitterionic glycolipid found in the cell wall of several Gram-positive bacterial strains, including *L. acidophilus*, which facilitates the adhesion, colonization, and invasion of cells by the bacteria [10,11]. The best studied form of LTA is composed of a polyglycerophosphate chain that is tethered to the membrane via a glycolipid anchor [12]. Studies indicate that LTA shares many of the inflammatory properties of lipopolysaccharide (LPS) via interactions with Toll-like receptors (TLRs) [13-16] which evoke diverse responses in innate cells through distinct signaling cascades [17].

Previously, we have demonstrated that deletion of the gene responsible for LTA biosynthesis in *L. acidophilus *NCFM diminishes this bacterium's capacity to stimulate the immune system; thereby suppressing pathogenic CD4^+^T cells in induced colitis [18,19]. To further investigate the role of LTA in inflammation, we engineered the NCK2031 strain in order to evaluate the effects, if any, of altered *s*urface *l*ayer *p*rotein (Slp) expression on LTA-induced pro-inflammatory signals and colitis.

## Methods

Materials Six to 8-week-old C57BL/6 were purchased from Jackson Laboratories (Bar Harbor, ME). Mice were maintained in microisolator cages under specific pathogen-free, *Helicobacter-*free conditions. Experiments were performed in an accredited establishment according to NIH guidelines in the Guide for Care and Use of Laboratory Animals (NIH-72-23), and animal protocols were approved by the local ethics committee. Dextran Sulfate Sodium (DSS) was obtained from MP Biochemicals (Solon, OH). Monoclonal antibodies for CD4, CD25, CD3, CD11c, CD11b, CD40, CD44, CD80, CD83, CD86, CD103, IL-10, IL-12, IFNγ, TNFα, HLA-ABC (R&D systems, Minneapolis, MN) and (BD, Franklin Lakes, NJ), CD1a (Dako, Carpentaria, CA), mouse and human GM-CSF and IL-4 were purchased from Invitrogen (Carlsbad, CA).

Generation of NCK2031 To generate an *L. acidophilus *NCFM isogenic mutant defective in all three *slp *genes (*slpA, slpB*, and *slpX*), the *slpB *(LBA0175) and *slpX *(LBA0512) genes were sequentially deleted in an NCFMΔ*upp *background host (NCK1909) using the *upp*-based counterselective gene replacement system [20]. Subsequently, attempts to insertionally inactivate the *slpA *gene (LBA0169) were made within the Δ*slpBX *double mutant (NCK2030) using a pORI-based gene knockout system [21]. The resulting Δ*slpBX *strain was deficient in SlpB and SlpX, but due to genetic instability of the insertion vector, NCK2031 continued to express SlpA (data not shown). Subsequently, wild-type *L. acidophilus *(NCK56), NCK2031, or LTA-deficient NCK2025 were propagated in de Man, Rogosa, and Sharpe broth (MRS, Difco) at 37°C for 15 hrs. The concentration of each *L. acidophilus *strain was adjusted to 1 × 10^9 ^CFU/ml based on OD600 readings that had previously been correlated with CFU numbers [22]. Each mouse was orally treated with 5 × 10^8 ^CFU bacterial strain; therefore 500 μl of 1 × 10^9 ^CFU/ml suspension was centrifuged, pelleted and then resuspended in 100 μl of PBS. Each mouse was then treated with 100 μl PBS (control group) or 5 × 10^8 ^CFU (of either *L. acidophilus *strain) orally in100 μl sterile PBS. Fecal pellets were collected from before, during, and for up to 8 days after *L. acidophilus *oral treatments. Each fecal pellet was resuspended in PBS (1:10 dilution, w/v). The suspension was then serially diluted and plated onto MRS agar containing Em (2 μg/mL). The homogenized material was serially diluted and plated onto MRS agar containing Em (2 μg/mL). The daily average CFU of the *L. acidophilus *strains in mouse feces were determined. For in vitro stimulation, bone marrow or human monocyte derived DCs were stimulated at a 1:1, 1:10, 1:100 ratio with live NCK2031, NCK56, NCK2025, or *S. aureus*-LTA. Subsequently the ratio of 1:1 was chosen, as this did not overwhelmingly activate DCs and therefore did not result in cell anergy or apoptosis. For the oral gavage of mice, each mouse received 5 × 10^8 ^CFU of NCK2031, NCK56, or NCK2025 in 100 μL of PBS.

Cell culture Immature human [22,23] or murine DCs [24] were treated with live *L. acidophilus *strains cells at 1:1 or with *S. aureus*-LTA (50 μg/mL) for 24 hrs. *L. acidophilus*- or *S. aureus*-LTA treated and untreated DCs (5 × 10^5^) were then stained and analyzed by BD FACSCaliber or a multicolor FACSCanto. Cell supernatants were also collected and analyzed using cytometric bead array kits (BD Biosciences). At least 1 × 10^4 ^gated events per condition were acquired. Analysis software (BD CellQuest) allowed for calculation of cytokine values in supernatants at pg/mL.

Immunofluorescence The colons of mice (n = 5/group) treated with live NCK2031, NCK56, NCK2025, *S. aureus*-LTA, or PBS alone were fillet-opened, rolled, and snap frozen at -80°C in Tissue-Tek O.C.T. (Sakura Finetek USA, Inc., Torrance, CA). Sections (5 μm) were cut, fixed in ice-cold methanol (-20°C) for 15 min and blocked with 1% BSA. Sections were then incubated overnight at 4°C with purified hamster anti-mouse CD11c (BD Biosciences) and rat anti-mouse IL-10 (BioLegend), and antibodies for IL-12, IFNγ, TNFα, CD8, and Foxp3, followed by washing twice with PBS and incubation with anti-hamster AlexaFluor 594 and anti-rat AlexaFluor 488 (Invitrogen) for 1 hr. Sections were then washed twice with PBS and incubated for 10 minutes with 4,6-diamidino-2-phenylindole dihydrochloride (DAPI, Invitrogen), washed with PBS two times and mounted with anti-fade mounting medium, as described previously [25]. Images were acquired using TissueGnostics Tissue/Cell High Throughput Imaging and Analysis System and analyzed using ImageJ software.

Flow cytometry Groups of mice (5 mice/group) were inoculated orally with NCK2031, NCK56, NCK2025, or *S. aureus*-LTA (5 × 10^8 ^CFU/100 μL of sterile PBS/mouse) or PBS alone. Mice were then sacrificed after 1, 3, or 7 days; single cells were isolated from the colons [18], MLNs, and spleens and stained with CD11c, CD11b, CD103, MHC II, CD40, CD80, CD86, F4/80, CD4, CD3, CD80, or CD44 [26]. Stained cells were then fixed, permeabilized, and stained with IL-12, TNFα, IFNγ or isotype antibodies. At least 1 × 10^5 ^gated events per condition were acquired. Data were acquired with BD FACSCanto II and analyzed using Tree Star FlowJo software.

DSS-Induced Murine Colitis Groups of C57BL/6 mice (n = 5/group) were inoculated orally with NCK2031, NCK56, NCK2025, or (5 × 10^8 ^CFU/100 μL PBS/mouse) for four consecutive days. In addition, a group of mice was treated with purified *S. aureus-*LTA (12.5 μg/μL) [27] for 5 days. All of these mice received one 5-day cycle of 3% DSS in drinking water, followed by 3 days of regular drinking water, with or without *S. aureus-*LTA, and then sacrificed on day 13. Acute colitis was observed after the first cycle of DSS in the non-inoculated group. Disease progression, including weight loss, diarrhea and fecal hemoccult blood positivity (FOB), was monitored throughout the study. Thereafter, mice were sacrificed and excised colon cross-sectional Swiss rolls were fixed in 10% formaldehyde and embedded in paraffin. Tissue sections (4 μm) were stained with hematoyxylin and eosin (H&E), and blindly scored, as described previously [28,29]. The grading, based on a scale from 0 to 28, takes into account the degree of inflammatory infiltrate, the presence of erosion, ulceration, or necrosis, and the depth and surface extension of the lesion.

## Results

### Immune stimulation by NCK2031

**Figure 1:**
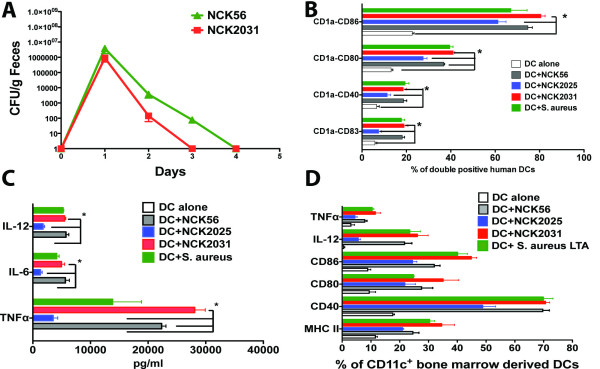
**Persistence of NCK56 and NCK2031 in the gut**. **(a) **Groups of mice (n = 3) were orally treated with (Em^r^)-resistant NCK56 or NCK2031 (5 × 10^8 ^CFU/20 μL/mouse). Fecal pellets were collected from before, during, and up to 8 days after bacterial oral treatments, and dissolved in 10% MRS. The homogenized materials were serially diluted and plated onto MRS agar containing Em (2 μg/mL). The daily average CFU of the *L. acidophilus *strains in the mouse feces were determined. These data are representative of at least three independent experiments. Activation of DCs by NCK2031.**(b, c, d) **Human or bone marrow derived DCs were activated with live NCK2031, NCK56, NCK2025, or *S. aureus *LTA (50 μg/ml) for 24 hrs at 37°C. Cells were harvested and phenotypically characterized by a FACSCaliber or a multicolor FACSCanto II. Cell supernatants were analyzed for cytokines by CBA using a FACSCaliber. Experiments were repeated at least five times with similar results. Data were statistically analyzed by one-way ANOVA, * P < 0.05 indicates statistically significant differences between the study groups.

To further characterize the role of LTA expression in inflammation, we attempted to generate *s*urface *l*ayer *p*rotein (Slp)-deficient strains using the *upp*-counterselective gene replacement system [20]. The resulting strain, NCK2031, showed no detectable expression of SlpB or SlpX while retaining LTA expression. Attempted insertional mutation in the *slpA *gene was unstable and spontaneous SlpA^+ ^revertants occurred in the population. Therefore, the phenotype of this mutant was defined as SlpA^+^B^-^X^-^LTA^+^. SDS-PAGE analyses confirmed the expression of SlpA only (at 46 kDa), and the absence of SlpB and SlpX within the NCK2031 culture (data not shown). To evaluate any in vivo physiological effects of NCK2031 and its colonization in the gut of bacteria treated mice, the persistence of erythromycin-resistant (Em^r^) NCK56 and Em^r ^NCK2031 strains in the colon were determined in C57BL/6 mice orally treated once with 5 × 10^8 ^CFU/mouse. Fecal pellets were collected the day before treatment and every day for up to 8 days after bacterial ingestion. Data reveal that mice cleared both NCK2031 and NCK56 (wild type) after 3 days, with significantly less NCK2031 excreted on days 2 and 3 (Figure 1a). Therefore, while the trends for clearance of the orally fed bacteria were similar, deletion of the SlpB and SlpX proteins appeared to accentuate clearance of the bacterium from the GI tract by one day, in vivo. Determining the time of bacterial clearance from the gut was essential to delineate the sampling time points used in the immunological studies.

To elucidate the physiological role of NCK2031 in dendritic cell (DC) activation, we co-cultured human monocyte derived DCs with NCK2031, NCK56, NCK2025, or purified *S. aureus*-LTA. Data revealed that the parent strain NCK56, the SlpA^+^B^-^X^-^LTA^+ ^strain NCK2031, and *S. aureus-*LTA profoundly stimulated human DCs, while the SlpA^+^B^+^X^+^LTA^- ^strain NCK2025 only marginally modified DC function (Figure 1b, c). Additionally, murine bone marrow derived DCs co-cultured with NCK2031 exhibited an up-regulation of MHC II, CD80, CD86, CD40, and increased production of intracellular interleukin (IL)-12 and TNFα when compared to DCs cultured with NCK56, NCK2025 or *S. aureus-*LTA (Figure 1d). These data indicate that LTA is not playing a regulatory role as seen in other studies [27,30], but rather, functions as a highly pro-inflammatory molecule when expressed on *L. acidophilus *[19].

### Activation of DCs, macrophages, and T cells by NCK2031

**Figure 2:**
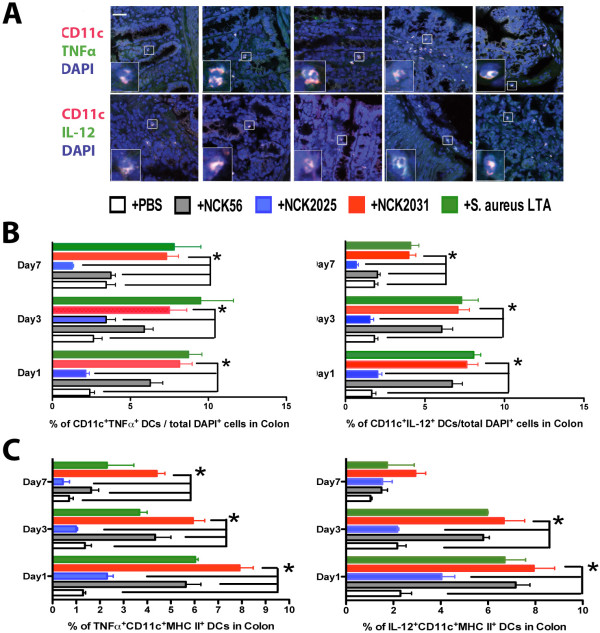
**Stimulation of cells by *L. acidophilus *strains**. Excised colons from untreated (PBS) mice (n = 5/group) and mice (n = 5/group) treated with NCK2031, NCK56, NCK2025, or *S. aureus *LTA were stained with CD11c, TNFα, IL-12, or CD4, IFNγ, and visualized by UV LSM. White size bar represents 20 μm. White arrows point to the co-localized cells in yellow dots. **(a) **Representation of CD11c^+^/TNFα^+^, CD11c^+^/IL-12^+ ^cells (yellow, merged red/green/blue). White size bar represents 50 μm. White arrows point to the co-localized cells in yellow dots. **(b) **Quantitative analyses of IL-12 and TNFα^+ ^by TissueGnostics Tissue/Cell High-Throughput Imaging and Analysis System. **(c) **Induction of colonic DC-activation by *L. acidophilus *expressing LTA. Single cells were derived from colons of the mice (n = 5/group) treated with NCK20331, NCK56, NCK2025, or PBS. Cells were stained with anti-CD11c, CD11b, CD103, MHC II, CD19, F4/80, IL-12, or TNFα antibodies. Live CD11c^+ ^cells that were F4/80^-^CD19^- ^were analyzed for their cytokine expression and expression of surface molecules (i.e., MHC II) by FACSCanto II. Experiments were repeated at least three times with similar outcomes.

**Figure 3:**
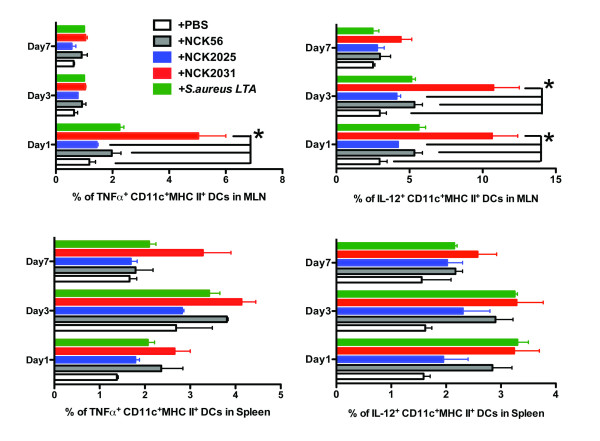
**Activation of mesenteric DCs by *L. acidophilus *strains**. **(a-b) **Single cells were derived from mesenteric lymph nodes (MLN) or spleens of the mice (n = 5/group) treated with NCK2031, NCK56, NCK2025, or PBS. Cells were stained with anti-CD11c, CD11b, CD103, MHC II, CD19, F4/80, IL-12, or TNFα antibodies. Live CD11c^+ ^cells that were F4/80^-^CD19^- ^were analyzed for their cytokine expression and expression of surface molecules (i.e., MHC II) by FACSCanto II. Experiments were repeated at least three times with similar outcomes.

To overcome the undesired effects of cell death induced by enzymatic reagents (i.e. collagenase) necessary for cell isolation we employed TissueGnostics fluorescent microscopy [18] and confocal microscopy (Figure 2a, 3a) to analyze the cell populations and their cytokine expression in the colons of bacteria treated mice. As demonstrated previously, a significantly higher percentage of CD11c^+^IL-10^+ ^DCs per total DAPI^+ ^cells were observed in the colons of mice treated with NCK2025 when compared to mice treated with NCK2031, NCK56, or *S. aureus-*LTA on days 1 and 3, but not by day 7 (data not shown). In contrast, IL-12 and TNFα production by colonic DCs was significantly higher in mice treated with NCK2031, NCK56, or *S. aureus*-LTA when compared to mice that were treated with NCK2025 or PBS (Figure 2b). To expand upon these findings, we studied the ability of the LTA-expressing strains to induce the stimulation of DCs, macrophages, CD4^+ ^and CD8^+ ^T cells in vivo. While no significant differences were seen in the expression of MHC II, CD40, CD80, or CD86 on CD11c^+^CD11b^+^DCs or CD11b^+^F4/80^+ ^macrophages (data not shown), cytoplasmic IL-12 and TNFα were significantly increased in CD11c^+ ^MHC II^+ ^DCs derived from the colons (Figure 2c), and mesenteric lymph nodes (MLNs) (Figure 3a), but not the spleens of mice that were treated with NCK2031, NCK56, or *S. aureus *-LTA versus PBS or NCK2025 (Figure 3b).

**Figure 4:**
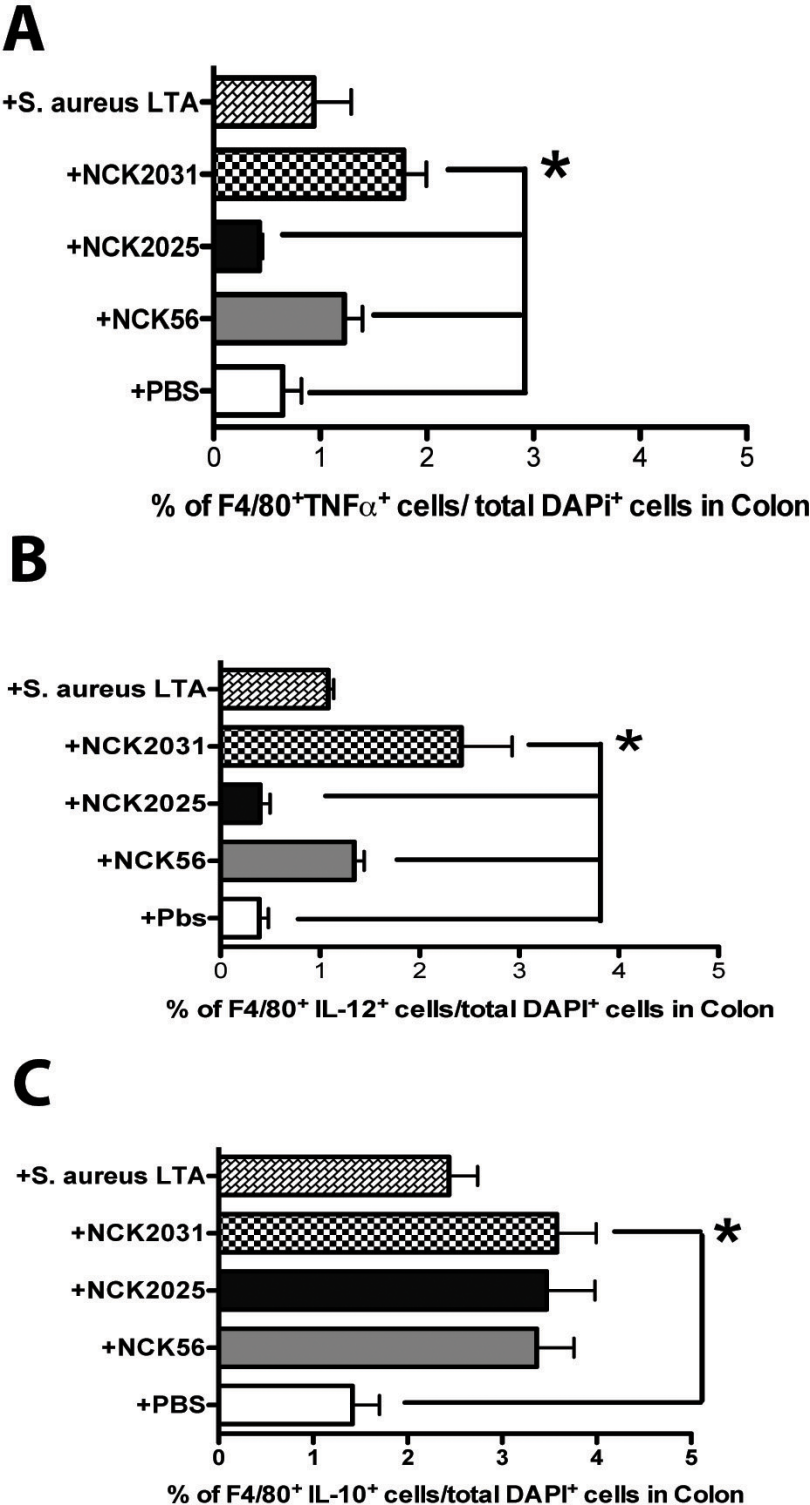
**Stimulation of colonic F4/80^+ ^macrophages**. **(a-c) **Quantitative analysis of **(a) **F4/80^+ ^TNFα^+^, **(b) **F4/80^+^IL-12^+ ^(c) F4/80^+^IL-10^+ ^in untreated (PBS) mice and in mice treated with NCK2031, NCK56, NCK2025, and *S. aureus*-LTA. Data were statistically analyzed by one-way ANOVA, * P < 0.05 indicates significance of the data between the study groups.

Recent studies have indicated that there is a significant recruitment of colonic F4/80^+ ^macrophages in IBD compared to that seen in normal mucosa [31]. Thus, we investigated the impact of the genetically modified strains versus the wild type NCK56 and *S. aureus*-LTA on the recruitment and activation of colonic F4/80^+ ^macrophages in vivo. Based on data generated by TissueGnostics, it was apparent that NCK2031, NCK56, and *S. aureus*-LTA significantly induced colonic F4/80^+ ^macrophages to produce IL-12 and TNFα; the expression of these cytokines was low in NCK2025-treated mice after 24 hrs (Figure 4a, b), but had leveled off by days 3 and 7 (data not shown). In contrast, IL-10 was highly expressed in NCK2031, NCK56, NCK2025, and *S. aureus-*LTA treated mice compared to untreated mice (Figure 4c). This observation may reflect the nature of the bacteria and their gene products, which can differentially stimulate these colonic cells to become either pro-inflammatory or regulatory. These data suggest that activation of intestinal cells and induction of inflammation may be triggered by the presence of LTA, as previously shown in lungs with airway inflammation [32,33].

**Figure 5:**
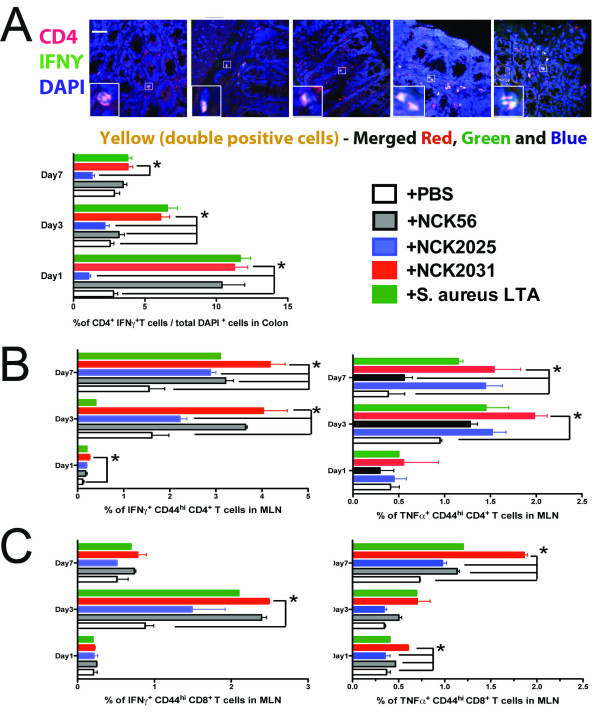
**Activation of CD4^+^(a, b) and CD8^+^T cells (c) by *L. acidophilus *expressing LTA**. After treatment of mice with PBS, NCK2031, NCK56, or NCK2025, lymphocytes were isolated from MLNs and spleens (not shown) after 1, 3 or 7 days and stained with live/dead, CD3, CD4, CD8, and CD44. Cells were fixed, permeabilized and intracellularly stained with TNFα, IFNγ and analyzed by FACSCanto II. Dead cells and doubles were excluded from analyses. Experiments were repeated at least three times with similar outcomes. Data were statistically analyzed by one-way ANOVA, * P < 0.05 indicates statistically significant differences between the study groups.

In addition, colonic CD4^+^T cells from mice treated with NCK2031, NCK56, or *S. aureus-*LTA expressed higher levels of IFNγ compared to mice treated with NCK2025 or PBS (Figure 5a). Treatment of mice with NCK2031, NCK56, and *S. aureus*-LTA also induced significantly increased expression of IFNγ and TNFα (Figure 5b), but not IL-17 (data not shown), in the CD4^+ ^T cells from the MLNs; a significant trend was not seen in the spleen (data not shown). The CD4^+ ^cells seemed to be less affected in the NCK2025-treated mice (Figure 5b).

It has recently been demonstrated that CD8^+^T cells efficiently prevent the generation of colitis [34,35] and play a critical role in controlling potentially pathogenic T cells [36] in IBD patients [37,38]. Such CD8^+^T cells with regulatory activity reside in the lamina propria (LP) of healthy individuals but not in the LP of patients with IBD [37,38]. Interestingly, the percentages of CD8^+^T cells in the colons of mice treated with NCK2031, NCK56, or *S. aureus-*LTA were significantly reduced (data not shown) when compared to NCK2025 treated mice. The majority of CD8^+^T cells in the colonic tissues from all groups of mice were IFNγ negative (data not shown), suggesting that colonic CD8^+^T cells are probably not involved in immune stimulation; rather, they exert immune regulation in the colon [36]. To demonstrate whether resident or recruited colonic CD8^+ ^T cells are Foxp3^+^, these cells were visualized by TissueGnostics fluorescent microscopy. Surprisingly, there were no significant differences in Foxp3^+ ^expression between the groups of the mice that were treated with the various *L. acidophilus *strains or *S. aureus*-LTA (data not shown). Further studies are required to study this cell type in colitis disease models. The activity of CD8^+ ^T cells within the MLN and spleen was also analyzed. NCK2031, NCK56, and *S. aureus*-LTA significantly stimulated MLN CD8^+^CD44^high ^T cells to produce IFNγ on day 3 and TNFα on day 7 (Figure 5c); no significant differences in the production of these cytokines were seen in the splenic CD8^+ ^T cells (data not shown). Collectively, these data indicate that the LTA-expressing strains, NCK2031 and NCK56, activate DCs and macrophages in a pro-inflammatory manner, which in turn, may directly elicit CD4^+ ^and CD8^+ ^T cell activation.

### Exacerbation of DSS-induced murine colitis by NCK2031 and *S. Aureus*-LTA

**Figure 6:**
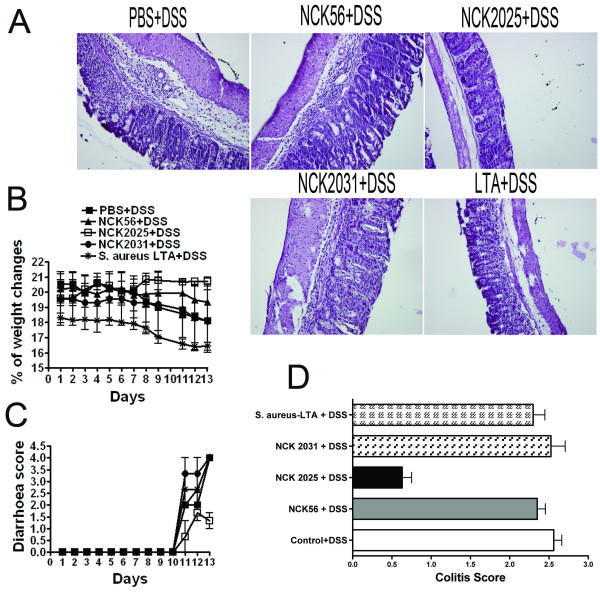
**DSS-induced murine colitis**. C57BL/6 mice (n = 5/group) were orally inoculated with NCK2031, NCK56, NCK2025 (5 × 10^8 ^CFU/100 μL/mouse) or PBS for 4 consecutive days. *S. aureus*-LTA (12.5 μg/μL) was dissolved in the drinking water and provided to the mice until day 13. These groups of mice were exposed to 3% DSS dissolved in the drinking water for 5 days followed by 7 days of plain water and assessed over time for colitis, including **(a) **hematoyxylin & eosin (H&E) staining, **(b) **weight loss, **(c) **diarrhea, and **(d) **disease activity index (DAI). Data are representative of at least three independent experiments.

To more thoroughly investigate the pro-inflammatory mechanisms induced by LTA, we utilized the DSS-mouse model for colitis [18]. Data show that DSS generated murine colitis in PBS-treated mice (Figure 6a-d). Mice began to lose weight after day 8 and developed severe diarrhea at days 10-13 (Figure 6b, c). To specifically address the role of LTA expression by NCK2031, mice were treated with NCK2031, NCK56, NCK2025 or *S. aureus-*LTA dissolved in drinking water. NCK2031, NCK56, or soluble *S. aureus-*LTA did not prevent the onset of colitis and the mice developed colitis similarly to non-treated mice (Figure 6a-d). In contrast, treatment with NCK2025 significantly prevented weight loss and reduced diarrhea (Figure 6), as previously documented [18]. Overall, the "Disease Activity Index" (DAI) was significantly increased for untreated animals and mice treated with NCK2031, NCK56, and *S. aureus-*LTA (Figure 6d). The colons from these mice exhibited ulcerated epithelium with significant inflammation confined to the mucosa, while colonic tissue obtained from mice treated with NCK2025 did not exhibit evidence of significant inflammation (Figure 6a).

## Discussion

Excessive stimulation of intestinal innate cells, including DCs, with commensal bacteria and/or their gene products results in immune dysfunction that can generate uncontrolled inflammation leading to tissue destruction and colitis [39]. Precise cellular and molecular mechanisms of these induced inflammatory immune responses in IBD remain poorly understood. Data so far indicate that chronic intestinal inflammation coincides with elevated levels of pro-inflammatory cytokines (i.e., IL-12, TNFα) [40], and the differentiation/activation of pro-inflammatory DC-subsets and pathogenic CD4^+^T cells [41]. Accordingly, promising studies show that inhibition of detrimental signals induced by stimulatory bacterial products mitigates IBD progression [42-49].

Together, our data show that LTA plays a critical role in the induction of the inflammatory responses and cannot prevent mice from developing colitis. Contrary to our findings, other studies have shown that lactobacilli-LTA induces regulatory signals (i.e., IL-10) via Erk1/2 signaling, resulting in anti-inflammatory mechanisms [30]. Other reports also indicate that commensal LTA protects mice from DSS-induced colitis and ameliorates impaired epithelial tight junctions [27,50]. This discrepancy may lay in the methodological approaches and reagents that were used in these studies. Here we demonstrate that LTA and LTA expressing bacteria posses the ability to stimulate innate immune component cells, including DCs and macrophages, which in turn, can trigger pathogenic IFNγ-secreting CD4^+ ^T cells in disease progression.

The role of IFNγ expressed in the colon is unclear, as it has been shown that this cytokine exerts protective features in different models of inflammation [51]. For example, in a murine model of multiple sclerosis, neutralization of IFNγ resulted in inflammatory immune responses [52]. Similarly, in a mouse model of IBD, IFNγ has been shown to suppress IL-23 [53], and collagen-induced arthritis is induced in the absence of IFNγ signaling [54]. However, in the models we have previously used for experimental DSS inducing epithelial injury (i.e., leaky mucosa) resulting in colitis or pathogenic CD4^+^CD45RB^high ^T cell transfer; IFNγ seems to be involved in the pathogenesis of the induced colitis. Additionally, IFNγ contributes to intestinal pathology and lethality in lipopolysaccharide-sensitization models of toxic shock syndrome (TSS) [55,56]. Further studies are needed to clearly elucidate the role of this cytokine in colitis and the timing of its production during disease progression.

Our results show that in vitro and in vivo, NCK2031 expressing LTA and purified *S. aureus*-LTA induce intestinal immune activation resulting in the production of TNFα and IL-12 and the development of pro-inflammatory cells such as CD11c^+^TNFα^+^IL-12^+ ^DCs and F4/80^+ ^macrophages that may contribute to an undesired CD4^+^IFNγ^+^TNFα^+ ^T cell pro-inflammatory response seen in colitis. Conversely, treatment with LTA-deficient NCK2025 elicited regulatory signals and induced less inflammation, as demonstrated here and previously [18,19]. We have previously demonstrated the involvement of SlpA of *L. acidophilus *NCFM in the immune regulation of DC functions [57]. Studies are underway to better delineate the physiological role of SlpA from this bacterium in vivo. Finally, an acute colitis characterized by bloody diarrhea, ulcerations and infiltrations with granulocytes can be induced in mice that are exposed for several days to significantly dissolved DSS polymers in drinking water [58,59]. As seen in Figure 6, we also could confirm such clinical signs when DSS was used to induce colitis. Additionally, our data clearly show not only the proinflammatory nature of LTA but also the significantly exacerbated disease progression in the colon of mice that received LTA in the drinking water. This data thus are in sharp contrast with the notion that LTA plays a regulatory and does posses the ability to mitigate DSS-induced colitis as described previously [27]. In summary, oral administration of DSS to mice induces an acute colitis, followed by a slow colonic regeneration of the epithelium with a concomitant exaggerated inflammation; furthermore LTA did not reduce the levels DSS-induced colitis or rapid healing of destructed epithelial tissues in our studies.

## Conclusions

Our data strongly suggest that directed alteration of two of the *L. acidophilus*-Slps did not ameliorate LTA-induced pro-inflammatory signals and subsequent colitis. Additionally, further studies are required to elucidate the role of *L. acidophilus*-Slps in the steady state of normal gut homeostasis and in diseases such as colitis and colon cancer where deregulated inflammation plays a critical role in disease progression. Finally, LTA did not mitigate DSS-induced colitis but induced further colonic inflammation.

## Abbreviations

DCs: Dendritic cells; LTA: Lipoteichoic acid; IL: Interleukin; TNFα: Tumor Necrosis Factor-alpha; Slps: Surface layer proteins; DAI: Disease Activity Index.

## Competing interests

The authors declare that they have no competing interests.

## Authors' contributions

MZ and MWK carried out all animal work, immunocytochemistry (i.e., high throughput and confocal imaging) and flow cytometry experiments. Additionally, MZ and MWK gathered the raw data and using statistical analyses and graphic programs compiled the data into meaningful information. MZ, MWK, JLO also contributed to writing the Methods section of the manuscript. MZ and MWK collected all of the graphics and formed the figures of the Manuscript. YJG, TK designed experiments and contributed to writing the Methods section. KS performed Slp proteome analysis on NCK2031 strain. MM designed experiments, discussed and wrote the manuscript. All authors have read and approved this manuscript.
